# Development of a robust flow cytometry-based pharmacodynamic assay to detect phospho-protein signals for phosphatidylinositol 3-kinase inhibitors in multiple myeloma

**DOI:** 10.1186/1479-5876-11-76

**Published:** 2013-03-23

**Authors:** Congfen Li, Chikara Takahashi, Liangxuan Zhang, Mahrukh Huseni, Basha Stankovich, Haider Mashhedi, Joanna Lee, Dorothy French, Jeff Eastham Anderson, Doris Kim, Kathy Howell, Matthew J Brauer, Marcin Kowanetz, Yibing Yan, Eric Humke, Allen Ebens, Garret Hampton, Mark R Lackner, Priti Hegde, Shidong Jia

**Affiliations:** 1 Genentech Inc, South San Francisco, CA, 94080, USA; 2 AllCells LLC, Emeryville, CA, 94608, USA

**Keywords:** Phosphatidylinositol 3-kinase, Pharmacodynamics, Biomarker, Multiple myeloma, Immunohistochemistry, Meso Scale Discovery, Flow cytometry, Clinical trial

## Abstract

**Background:**

The phosphatidylinositol 3-kinase (PI3K) pathway plays an important role in multiple myeloma (MM), a blood cancer associated with uncontrolled proliferation of bone marrow plasma cells. This study aimed to develop a robust clinical pharmacodynamic (PD) assay to measure the on-target PD effects of the selective PI3K inhibitor GDC-0941 in MM patients.

**Methods:**

We conducted an *in vitro* drug wash-out study to evaluate the feasibility of biochemical approaches in measuring the phosphorylation of S6 ribosomal protein (S6), one of the commonly used PD markers for PI3K pathway inhibition. We then developed a 7-color phospho-specific flow cytometry assay, or phospho flow assay, to measure the phosphorylation state of intracellular S6 in bone marrow aspirate (BMA) and peripheral blood (PB). Integrated mean fluorescence intensity (iMFI) was used to calculate fold changes of phosphorylation. Assay sensitivity was evaluated by comparing phospho flow with Meso Scale Discovery (MSD) and immunohistochemistry (IHC) assays. Finally, a sample handling method was developed to maintain the integrity of phospho signal during sample shipping and storage to ensure clinical application.

**Results:**

The phospho flow assay provided single-cell PD monitoring of S6 phosphorylation in tumor and surrogate cells using fixed BMA and PB, assessing pathway modulation in response to GDC-0941 with sensitivity similar to that of MSD assay. The one-shot sample fixation and handling protocol herein demonstrated exceptional preservation of protein phosphorylation. In contrast, the IHC assay was less sensitive in terms of signal quantification while the biochemical approach (MSD) was less suitable to assess PD activities due to the undesirable impact associated with cell isolation on the protein phosphorylation in tumor cells.

**Conclusions:**

We developed a robust PD biomarker assay for the clinical evaluation of PI3K inhibitors in MM, allowing one to decipher the PD response in a relevant cell population. To our knowledge, this is the first report of an easily implemented clinical PD assay that incorporates an unbiased one-shot sample handling protocol, all (staining)-in-one (tube) phospho flow staining protocol, and an integrated modified data analysis for PD monitoring of kinase inhibitors in relevant cell populations in BMA and PB. The methods described here ensure a real-time, reliable and reproducible PD readout, which can provide information for dose selection as well as help to identify optimal combinations of targeted agents in early clinical trials.

## Background

Multiple myeloma (MM), also known as myeloma or plasma cell myeloma, is a B cell neoplasm characterized by monoclonal plasma cell expansion localized in the bone marrow. MM is the second most prevalent hematologic malignancy after non-Hodgkin’s lymphoma. It represents approximately 1% of new cancer cases and 2% of cancer deaths in the United States, with an estimated 21,700 new cases and 10,710 cancer deaths in 2012 [1]. In recent years, the overall survival of myeloma patients has improved to 7 to 8 years due to the widespread use of several novel agents (Bortezomib, lenalidomide and thalidomide) and the incorporation of autologous hematopoietic stem cell transplantation. However, the etiology and the mechanism of disease development of MM are still incompletely understood.

The pathogenesis of MM involves multistep genetic and microenvironmental changes, including chromosome abnormalities (translocations and deletion) and de-regulation of oncogenic signaling pathways [1]. The activation of growth factor pathways such as interleukin 6 (IL-6) and insulin-like growth factor I (IGF-I) have been found to activate the downstream phosphatidylinositol 3-kinase (PI3K)-AKT signaling cascade in MM [2]. In fact, late-stage MM tumors exhibit dramatically enhanced AKT activation [3], and inhibition of the PI3K-AKT pathway has been effective in preclinical studies of MM [2-6]. Decades of studies have provided the rationale for testing PI3K inhibitors, such as GDC-0941, in cancer prevention and treatment [7-9]. Currently, small molecule inhibitors targeting PI3K and AKT (Perifosine) pathways are being evaluated in clinical trials of MM [10].

Robust tumor PD assays would be useful for the clinical evaluation of GDC-0941 in myeloma patients. Among others, IHC is the current gold-standard PD assay routinely used in clinical trials, while Meso Scale Discovery (MSD) is one of the most sensitive biochemical assays in PD studies. Reduced phosphorylation of downstream proteins, including S6, is a commonly used proof-of-mechanism of PI3K pathway inhibition [8,11]. GCD-0941 is a potent oral pan-PI3K inhibitor that has demonstrated reduced phosphorylation of downstream proteins including S6 ribosomal protein (pS6) in tumor cells by IHC in Phase I studies of patients with solid tumors [12]. From a tumor PD perspective, there are intrinsic caveats with IHC or MSD assays to assess the protein phosphorylation of S6 in MM [10], a blood cancer whose tumor cells reside within the bone marrow with variable tumor content between 5-95% in newly diagnosed patients.

Protein phosphorylation is a transient event that can be preserved by formalin-containing fixatives and captured by flow cytometry-based phospho flow, an assay particularly suitable for studying the phosphorylation status in a complex cell population such as BMA or PB. In this study we adapted a phospho flow cytometric method to develop a robust phospho flow-based PD assay for the clinical evaluation of PI3K inhibitors in MM, monitoring protein phosphorylation and pathway modulation in the relevant cell types.

## Methods

### Materials

Test compound GDC-0941 was synthesized at Genentech. Stock solutions of GDC-0941 were prepared in DMSO and diluted in the indicated culture medium for the treatment of cells or directly added into bone marrow aspirate (BMA) and/or peripheral blood (PB) (final concentration of DMSO, <0.1%).

### Cell culture

MM1s and RPMI 8226 cell lines were maintained in RPMI 1640 media supplemented with 10% fetal bovine serum (FBS) and 1 mM glutamine (Sigma-Aldrich) at 37°C in a humidified incubator containing 5% CO_2_. Fresh BMA and PB were obtained from healthy donors at Genentech or from myeloma patients at a local clinic with appropriate informed consents for exploratory biomarker evaluation. Whole blood or BMA were collected in heparinized vacutainers, and transported to the laboratory within 3 hours of collection. Where indicated, samples (tumor cells, BMA, or PB) were treated with DMSO or GDC-0941 *ex vivo* at 37°C in a humidified incubator with 21% O_2_ and 5% CO_2_.

### Phospho-specific flow cytometry

After incubation, the samples (tumor cells, BMA, PB) were immediately fixed for 10 minutes by adding 1× lyse/fix buffer (BD Biosciences) at room temperature (RT), and then permeabilized with cold 100% methanol on ice for 10 min. After washing with phosphate buffered saline (PBS) and FBS based staining buffer, approximately one million cells per tube were stained for 30 minutes in the dark with an antibody cocktail prior to flow cytometric analysis.

Blood lineage-specific surface CD markers were stained with the following antibodies: CD45 Alexa 700 (HI30), CD14 Alexa 488 (M5E2), CD38 V450 (HB7), CD138- PE (MI15), CD20 PerCP-Cy5.5 (2H7), CD3 PE- Cy7 (SKY7), (BD Biosciences). Intracellular phospho-protein was stained using a specific monoclonal antibody against pS6 Ser235/236 Alexa 647 (Cell Signaling). The primary antibodies were diluted at the optimal dilution according to the manufacturer’s instructions.

Appropriate isotype controls for cell surface markers and rabbit (DA1E) mAb IgG XP® Isotype Control (Alexa Fluor® 647 Conjugate) for pS6 were used to facilitate the gating of specific cell populations of interest. QC of the FACSCAntoII instrument was preformed on each day of the study. A set of BD anti-mouse Ig, k/negative control (FBS) CompBeads was used to optimize the fluorescence compensation setting necessary for multicolor flow cytometric analyses. At least 10,000 cell events were collected and analyzed on a BD FACSCanto II system (BD Biosciences). Flow cytometry analysis was carried out using FACSDiva Software and Prism 4.0 (GraphPad).

To minimize day-to-day variation in cytometer settings and perform daily QC, CST beads with fluorescence in all channels (BD Biosciences) were tested at the beginning of each acquisition run. Fluorescence values varied by <10% of target values. For the assay reproducibility study, BMA and PB samples from the same donor were separated into multiple replicates for phospho flow analysis.

### MSD

Cells were washed with phosphate buffered saline (PBS), lysed with MSD lysis buffer and incubated on ice for 30 minutes. Soluble proteins were collected by spinning at 20000g for 10 minutes. Equal amounts of protein (20 ug) were added into each well of pS6 (S235/236)/total S6 MSD plate (Cat# K150DFD-3; Meso Scale Discovery, LLC), and MSD assays were performed according to the manufacturer’s instructions.

### IHC staining and pS6 quantification

MM1s cells (8 × 10^7^ cells) were treated with GDC-0941 for 2 hrs at each of the indicated concentrations. Cells were washed once with cold PBS and cell pellets were fixed in buffer neutralized formalin overnight prior to paraffin embedding.

Immunohistochemistry (IHC) was performed on 4 μm thick formalin-fixed paraffin embedded tissue sections mounted on glass slides. All IHC steps were carried out on the Ventana Discovery XT (Ventana Medical Systems; Tucson, AZ) autostainer. Pretreatment was done with Cell Conditioner 1, standard time. Primary antibody, anti-phospho-S6 (Rabbit polyclonal, 2211L, Cell Signaling Technologies, Cambridge, MA) was used at a concentration of 0.26 μg/ml and was incubated on slides for 32 minutes at 37°C, followed by incubation with Ventana Ultramap-HRP for 16 minutes. Ventana DAB and Hematoxylin II were used for chromogenic detection and counterstain.

Whole slide images were acquired by the Olympus Nanozoomer automated slide scanning platform (Hamamatsu, Bridgewater, NJ) at 200× final magnification. Scanned slides were analyzed in the Matlab software package (version R2011b by Mathworks, Natick, MA) as 24-bit RGB images. Cellular area was identified using intensity thresholding and standard morphological filtering. The brown DAB-specific staining was identified using a blue-normalization algorithm as described by Brey et al. [13]. The optical density of DAB staining was calculated using the Beer-Lambert law, absorbance = −log (transmitted light / incident light), on DAB positive areas only.

### Statistical analysis

The concentration of drug resulting in 50% maximal inhibitory concentration (IC50) was determined using Prism software (GraphPad). All statistical analyses were conducted with the Student *t* test and are represented as mean ± SD. One asterisk indicates p < 0.05, and two asterisks indicate p < 0.01.

## Results

### Evaluation of IHC and MSD assays in myeloma cell lines

**Figure 1:**
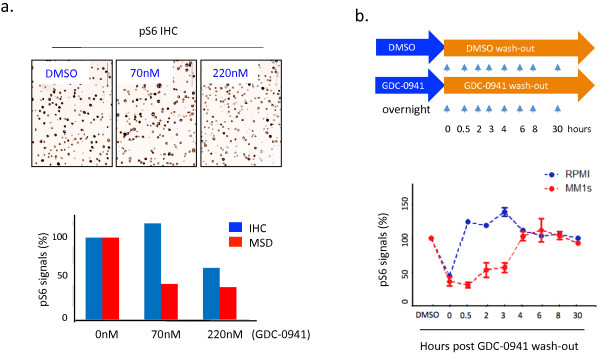
**Effects of drug wash-out on protein phosphorylation.** (**a**). MM1s cells were treated with DMSO or GDC-0941 at indicated doses for 2 hours. Cell pellets were formalin fixed and paraffin embedded, and sectioned for pS6 staining (top). The level of pS6 was quantified by IHC and MSD assays (bottom). (**b**). Two MM cell lines (MM1s and RPMI8226) were treated with DMSO or GDC-0941 (200nM) overnight, washed three times and cultured in regular culture medium in the absence of GDC-0941 or DMSO. Cells were harvested at indicated times for MSD biochemical assay. The ratio of the normalized pS6/tS6 between GDC-0941 and DMSO treatments was used to calculate the percentage of pathway modulation. Data shown (**a**-**b**) are representative of at least three independent studies.

IHC is a popular assay in clinical PD biomarker studies. To assess the sensitivity of the IHC assay, we conducted a drug titration study by treating MM1s myeloma cells with various amount of GDC-0941, and measured the status of pS6 by IHC and MSD, a highly sensitive assay for quantification. Figure 1a (top) shows the level of pS6 staining in MM1s cells in response to GDC-0941. Quantification analysis indicated that MSD could detect pS6 down-regulation at 70nM of GDC-0941 compared to the higher dose (220nM) detected by IHC (Figure 1a, bottom). Thus, IHC is not a very sensitive assay compared to MSD, where the down-regulation of pS6 revealed by IHC would strongly support the conclusion of PD response, but a lack of change by IHC does not indicate the lack of PD response in clinical samples.

In a typical MM tumor PD study, isolation of tumor cells from heterogeneous groups of cell populations in BMA can take up to 2–3 hours with purification of bone marrow mononuclear cells (BM MNC) and further bead-based cell isolation. To determine the impact of tumor cell isolation process on the integrity of phospho protein signals, we performed a drug wash-out study with GDC-0941 using the cultured myeloma tumor cell lines and evaluated the extent of phosphorylation change of S6, a downstream target of PI3K signaling (Figure 1b, top). Remarkably, our MSD results revealed that upon drug washout there was a cell line-specific recovery of S6 phosphorylation over time. RPMI cells quickly restored the previously reduced pS6 within half an hour post drug wash-out, while MM1s cells maintained knockdown of pS6 for up to 3 hours (Figure 1b, bottom). The quick recovery of pS6 observed herein suggests a similar scenario could potentially occur in clinical BMA samples that undergo cell isolation and could lead to false data interpretation.

Together, the IHC assay was less sensitive in terms of signal quantification while the biochemical approach (MSD) was less suitable to assess PD activities due to the undesirable impact associated with cell isolation on the protein phosphorylation in tumor cells.

### Development of a pS6 phospho flow assay

**Figure 2:**
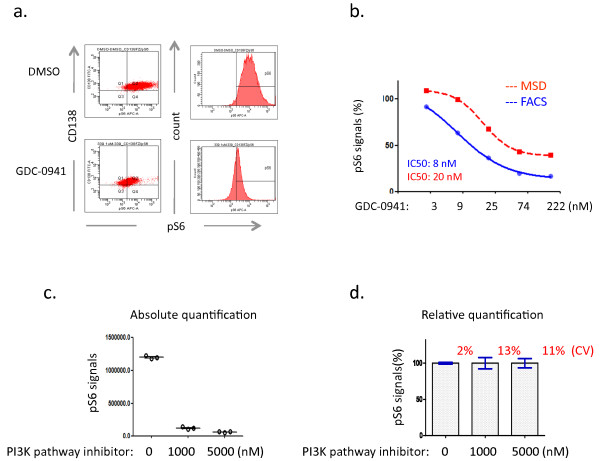
**Development of phospho flow assay.** (**a**). PD response revealed by phospho flow analysis in MM1s cells. Note the peak shift of pS6 on the histogram in the GDC-0941-treated MM1s cells compared to DMSO. Baseline is defined based on isotype staining. (**b**). Cross-platform comparison between MSD and phospho flow cytometry. iMFI methodology is used to calculate the pS6 signals (**c and d**). Intra-assay variation studies demonstrate the absolute quantification of pS6 signals (**c**) and relative quantification (**d**), highlighting the coefficient of variation (CV). Note that MM1s cells were treated with DMSO or a PI3K pathway inhibitor (tool compound) for 2 hours in the above studies. Data shown (**a**-**d**) are representative of at least three independent studies.

Here we developed a phospho flow assay to assess pS6 pathway modulation in response to GDC-0941. Figure 2a shows the shift of the high level of pS6 in tumor cells towards baseline in response to GDC-0941. Similarly, robust pS6-based PD pathway down-regulation has been observed with other PI3K pathway inhibitors, but not with non-PI3K pathway inhibitor when tested in this study (data not shown). Quantification methodology is a critical issue in phospho flow data analysis. We re-evaluated the commonly used methods in phospho flow studies, specifically percentage of phosphorylation-positive cells (percentage) or mean fluorescence intensity (MFI), and demonstrated that percentage or MFI does not accurately quantify fold changes in protein phosphorylation upon drug treatment (Additional file 1: Figure S1). We adopted a metric termed the integrated MFI (iMFI) [14,15], defined as the percentage of pS6-positive tumor cells multiplied by MFI of pS6-positive tumor cells, to calculate the total functional phosphorylation signals in subsequent PD studies.

To assess the sensitivity of phospho flow, we designed a drug-titration study where MM1s cells were first treated with various amounts of GDC-0941, and then split for phospho flow analysis or MSD. Figure 2b shows a strong correlation between the two platforms, demonstrating comparable sensitivity for monitoring pathway modulation with an IC50 of 8nM by phospho flow and 20nM by MSD. Intra-assay variation studies showed that the phospho flow assay is highly reproducible with only minor variability (Figure 2c and 2d).

### Development of tumor PD and surrogate PD biomarkers

**Figure 3:**
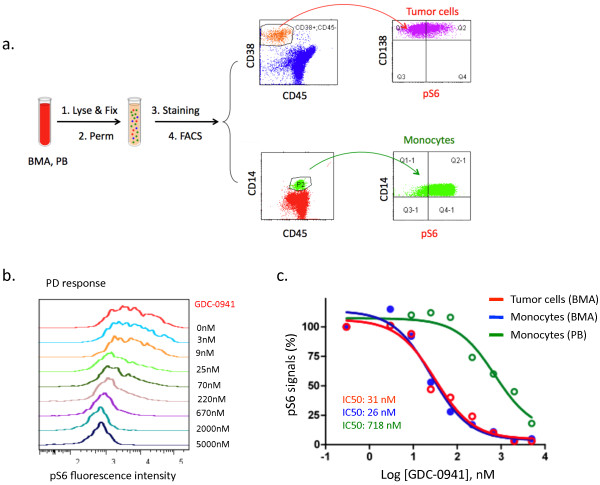
**Development of tumor PD and surrogate PD biomarkers using phospho flow assay.** (**a**). Gating strategy of tumor and surrogate tissues in pS6 phospho flow study using BMA and PB obtained from myeloma patients. Representative scheme is shown for myeloma tumor cells (CD45-; CD38+; CD138+) in BMA and monocytes (CD45+; CD14+) in PB and BMA. FACS, i.e., fluorescence-activated cell sorting, is a specialized type of flow cytometry. (**b** and **c**). GDC-0941 dose-dependent PD response. Patient-derived BMA and PB were treated with GDC-0941 at indicated doses *ex vivo* for 2 hours. Phospho flow analysis demonstrated the GDC-0941 dose-dependent tumor PD response in histogram (**b**) and % of pS6 baseline signals in tumor cells and monocytes (**c**). Data shown are representative of at least three independent studies.

To take advantage of the resolving power of simultaneous intracellular staining of the phospho-epitope and surface staining with immunophenotypic markers, we further developed a 7-color antibody panel that allows for PD monitoring of S6 protein phosphorylation in tumor and surrogate tissues using BMA and/or whole blood (Table 1). We performed a dose-titration study using MM patient-derived BMA and PB. In addition to myeloma tumor cells and monocytes as shown in Figure 3a, we also gated on B cells (CD45+; CD20+) and T cells (CD45+; CD3+). Phospho flow analysis showed that pS6 was highly expressed in tumor cells and monocytes in contrast to the barely detected pS6 in B and T cells (Figure 3a, Table 1). Both tumor cells and monocytes exhibited a dose-dependent pS6 down-regulation in response to GDC-0941 at a clinically achievable concentration (Figure 3b and 3c). Of note, tumor cells and BMA-derived monocytes were more sensitive when compared to blood monocytes (IC50: 31nM in tumor cells, 26nM in BMA monocytes, and 718nM in PB monocytes) (Figure 3c).

**Table 1:** Basal levels of pS6 in MM tumor and immune cells

**Cell type**	**CD marker**	**Location**	**pS6**	**PD response**
Tumor cells	CD45-; CD38+; CD138+	BMA	+++	Robust
Monocytes	CD45+; CD14+	PB, BMA	+++	Moderate
B cells	CD45+; CD20+	PB, BMA	-	N/A
T cells	CD45+; CD3+	PB, BMA	-	N/A

### Development of a one-shot sample handling protocol for clinical trials

**Figure 4:**
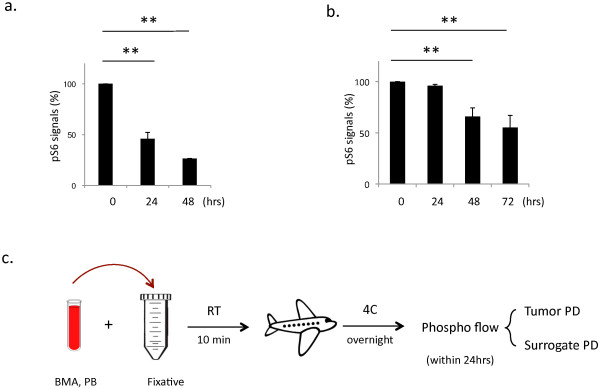
**Development of one-shot sample handling protocol.** (**a**). Ambient storage conditions impair the pS6 status over time. Y-axis refers to % of pS6 baseline signals. (**b**). The one-shot sample handling protocol provided well-preserved pS6 signals for 24 hrs. (**c**). An overview of the phospho flow-based PD study proposed for clinical trials. Data shown (**a**-**b**) are representative of at least three independent studies.

The stability of phospho-epitopes is of particular concern when applying phospho flow in the clinic. MM BMA and blood samples in clinical trials are often freshly collected and directly shipped under ambient conditions for downstream studies. To assess whether the stability of pS6 in unfixed cells is affected by the ambient conditions, we performed a tumor cell spike-in study by adding tumor cells into PB and stored them at room temperature. Of note, phospho flow analysis demonstrated that the basal level of pS6 dropped dramatically over time in both tumor cells (Figure 4a) and monocytes (data not shown), suggesting caution of the handling of unfixed cells under ambient conditions and emphasizing the value of immediate cell fixation to preserve the phosphorylation of S6.

Sample collection and handling is more complicated in clinical laboratories than in preclinical settings. Every step of sample handling in clinical laboratories may increase the risk of a false PD readout. To simplify the process, we developed a one-shot sample handling procedure after testing 15 conditions of sample fixing and handling. The final procedure involves the following steps: transfer BMA or whole blood into a tube containing lysis/fixation buffer and incubate for 10 minutes at RT, followed by immediate storage/shipment at 4°C without any need for centrifugation, aspiration, or washing procedures. When compared to results found with fresh tumor samples, the pS6 signal was well preserved for at least 24 hours in fixative without noticeable loss in signal strength (Figure 4b). Thus, fixation of BMA and PB at RT followed by storage and shipment at 4°C produced optimal results for pS6 staining. An overview of the optimal clinical phospho flow-based PD assay is presented in Figure 4c.

## Discussion

Phospho flow can provide rich information in monitoring PI3K signaling cascades in complex cell populations such as BMA and PB, eliminating the need for any cell purification used in traditional biochemical studies. Following the elegant work by Nolan’s group [16], this study has further optimized phospho flow PD assay from the perspective of clinical applications. Specifically, immediate fixation of BMA or PB provides the best preservation of protein phosphorylation in a more physiological environment considering the nature of myeloma to pathogenesis, which is heavily influenced by tumor-stroma interaction [1]. A case in point is the enhanced drug sensitivity in BM monocytes that could be associated with cytokine stimulation as well as stromal and/or microenvironment bone marrow factors that are absent or present in low concentrations in PB. The one-shot sample handling procedure is extremely attractive because of its simplicity and reproducibility, clinical applicability, thereby minimizing changes associated with cell manipulation bias while maximizing the accuracy of PD readouts. These attributes are particularly important for multicenter clinical trials. The incorporation of the 7-color antibody panel into a single staining tube further enables multi-parameter data acquisition and analysis, reducing experimental time and inter-experiment variability, while allowing PD analysis in multiple cell types. iMFI-based data analysis provided an accurate quantification of fold changes in phosphorylation in response to GDC-0941. Finally, cross-platform comparison revealed a strong correlation of assay sensitivity between phospho flow and MSD, whereas IHC assay was less sensitive in terms of quantification.

Harvesting quality BMA (pre- and post- drug treatment) is a challenge in clinical trials. In this regard, PB provides an easily obtainable source to serially monitor S6 phosphorylation, generating reliable and reproducible PD readouts from monocytes and sometimes circulating tumor cells. The surrogate PD assay developed here complements the use of a platelet rich plasma (PRP) assay, another sensitive surrogate PD biomarker assay currently used in PI3K clinical trials. Despite their threshold discrepancy (Figure 3c), PB monocyte-based surrogate biomarkers (IC50: 718nM) could be informative given that the clinically achievable exposures of GDC-0941 are between 800–3000 nM at maximum plasma concentration (Cmax).

We envision that phospho flow and its derivative CyTOF, which merges flow cytometry with mass spectrometry, potentially have more general applications in oncology biomarker studies, providing solutions to address the therapeutic effects in heterogeneous cell populations that are often difficult to study biochemically. The protocol outlined here serves as a starting point for other phospho-epitopes, though challenges remain in that the approach will require extensive optimization of both the protocol and the reagents used to qualify additional endpoints for emerging clinical applications.

## Conclusions

We developed a robust PD assay and evaluated tumor and surrogate PD biomarkers for the clinical evaluation of GDC-0941 in MM. To our knowledge, this is the first report of an easily implemented clinical PD assay that incorporates an unbiased one-shot sample handling protocol, all (staining)-in-one (tube) phospho flow staining protocol, and an integrated modified data analysis for PD monitoring of kinase inhibitors in relevant cell populations in BMA and PB. The tumor and surrogate PD biomarkers described here may provide useful information for dose selection and confirm pathway modulation in response to PI3K inhibitor treatment, as well as potentially identify optimal combinations of targeted agents in early clinical trials.

## Abbreviations

BMA: Bone marrow aspirate; BM MNC: Bone marrow mononuclear cells; Cmax: Maximum plasma concentration; FBS: Fetal bovine serum; FMO: Fluorescence minus one; IL-6: Interleukin-6; IGF-I: Insulin-like growth factor-I; iMFI: Integrated mean fluorescence intensity; MFI: Mean fluorescence intensity; MM: Multiple myeloma; MSD: Meso Scale Discovery; PB: Peripheral blood; PBS: Phosphate buffered saline; PI3K: Phosphatidyl inositol 3-kinase; PD: Pharmacodynamic; PRP: Platelet-rich plasma; pS6: Phosphorylated S6; RT: Room temperature; S6: S6 ribosomal protein

## Competing interests

The authors claim no potential competing interests.

## Authors’ contributions

CL conceived and carried out the experiments; CT conceived and carried out the experiments; LZ conceived and carried out the experiments; MH helped to develop the assay; BS helped to develop the assay; HM helped with experiment set-up; JL conducted pilot drug wash-out study; DF conducted pathology analysis; JEA conducted the IHC imaging quantification; DK helped to develop the assay, KH helped with experiment set-up and conceived the study; MB participated in data analysis; MK helped with experiment set-up; YY provided PI3K clinical data; EH participated in its design; AE participated in its design; GH participated in its design; ML participated in its design and coordination; PH participated in its design and helped to develop this assay; SJ supervised the study and wrote the paper with input from co-authors. All authors read and approved the final manuscript.

## Supplementary Material

Additional file 1: Figure S1 Evaluation of percentage, MFI, and iMFI by phospho flow. In this hypothetical study, cells were treated with DMSO or GDC-0941. Phospho flow revealed two types of PD responses in Scenario 2 and 3. The average amount of pS6 fluorescence intensity is labeled on each cell (circle). X axis refers to the fluorescence intensity of pS6, and y axis the CD marker. Click here for file
