# Supplementary material for: Development of a robust flow cytometry-based pharmacodynamic assay to detect phospho-protein signals for phosphatidylinositol 3-kinase inhibitors in multiple myeloma
Source: J Transl Med. 2013 Mar 23;11:76. doi: 10.1186/1479-5876-11-76 (PMC3623880; doi:10.1186/1479-5876-11-76)
Supplement: Additional file 1: Figure S1 — Evaluation of percentage, MFI, and iMFI by phospho flow. In this hypothetical study, cells were treated with DMSO or GDC-0941. Phospho flow revealed two types of PD responses in Scenario 2 and 3. The average amount of pS6 fluorescence intensity is labeled on each cell (circle). X axis refers to the fluorescence intensity of pS6, and y axis the CD marker. [file 1479-5876-11-76-S1.pdf]

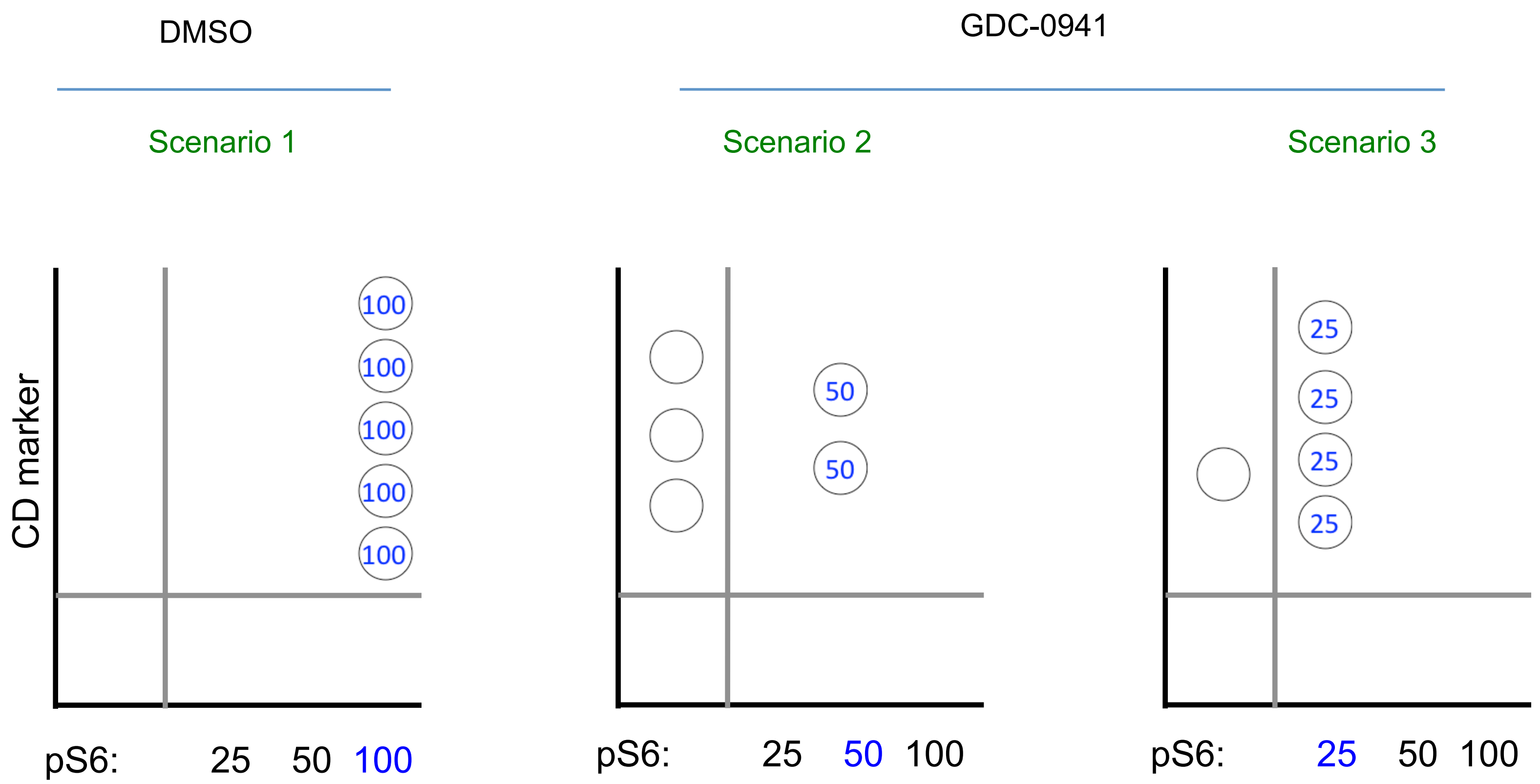

Supplementary Figure 1. Evaluation of percentage, MFI, and iMFI by phospho flow. In this hypothetical study, cells were treated with DMSO or GDC-0941. Phospho flow revealed two types of PD responses in Scenario 2 and 3. The average amount of pS6 fluorescence intensity is labeled on each cell (circle). X axis refers to the fluorescence intensity of pS6, and y axis the CD marker.
